# Supplementary material for: Undiagnosed hypertension and associated factors among bank workers in Bahir Dar City, Northwest, Ethiopia, 2020. A cross-sectional study
Source: PLoS One. 2021 May 27;16(5):e0252298. doi: 10.1371/journal.pone.0252298 (PMC8158901; doi:10.1371/journal.pone.0252298)
Supplement: S1 Questionnaire — (DOCX) [file pone.0252298.s002.docx]

**Questionnaires**

Structured questionnaires to assess the prevalence of undiagnosed hypertension and associated factors among bank workers in Bahir Dar city, 2020 G.C.

Data collector full name-----------------------------sign--------------- Date------------------

| **Instruction:** - I request you kindly to go through each question and encircle your answer. | | |
| --- | --- | --- |
| **Part I. Socio demographic characteristics** | | |
| **No** | **Variable** | **Response** |
| 101 | Age in year | -------------- years old |
| 102 | Sex | 1. Male 2. Female |
| 103 | Marital status | 1. Never married 2. Married 3. Divorced 4. Widowed |
| 104 | Religion | 1. Orthodox Christian 2. Muslim 3. Catholic 4. Protestant 5. Other specify--------------- |
| 105 | Educational level? | -------------------- |
| 106 | Your job description in this bank? | -------------------- |
| 107 | Working experience? | -------------------Months/Years |
| **Part II. Knowledge towards Hypertension** | | |
| **A. Knowledge towards the causation of Hypertension** | | |
| 201 | Eating diet rich in salt can cause Hypertension. | 1. Yes 2. No 3. Don’t know |
| 202 | Being overweight can cause Hypertension. | 1. Yes 2. No 3. Don’t know |
| 203 | Anxiety or anger can cause Hypertension. | 1. Yes 2. No 3. Don’t know |
| 204 | Too much drinking of alcohol can cause Hypertension. | 1. Yes 2. No 3. Don’t know |
| 205 | Smoking cigarette can cause Hypertension. | 1. Yes 2. No 3. Don’t know |
| 206 | Hypertension will occur genetically. | 1. Yes 2. No 3. Don’t know |
| **B. knowledge towards the signs and symptoms of Hypertension** | | |
| 207 | Headache is the symptoms of Hypertension. | 1. Yes 2. No 3. Don’t know |
| 208 | Dizziness is the symptoms of Hypertension. | 1. Yes 2. No 3. Don’t know |
| 209 | Shortness of breath is the symptoms of Hypertension. | 1. Yes 2. No 3. Don’t know |
| 210 | Palpitation is the sign and symptoms of Hypertension. | 1. Yes 2. No 3. Don’t know |
| **C. Knowledge towards prevention of Hypertension** | | |
| 211 | Exercising regularly could prevent Hypertension. | 1. Yes 2. No 3. Don’t know |
| 212 | Monitoring of blood pressure is very important in order to prevent high blood pressure. | 1. Yes 2. No 3. Don’t know |
| 213 | Reducing stress level could prevent Hypertension. | 1. Yes 2. No 3. Don’t know |
| 214 | Eating fruits and vegetables could prevent Hypertension. | 1. Yes 2. No 3. Don’t know |
| 215 | Reducing the amount of salt intake could prevent Hypertension. | 1. Yes 2. No 3. Don’t know |
| **D. Knowledge towards complication of Hypertension** | | |
| 216 | Hypertension con cause heart diseases, if left untreated. | 1. Yes 2. No 3. Don’t know |
| 217 | Hypertension can cause visual impairment, if left untreated. | 1. Yes 2. No 3. Don’t know |
| 218 | Hypertension can cause Stroke, if left untreated. | 1. Yes 2. No 3. Don’t know |
| 219 | Hypertension can cause kidney failure, if left untreated. | 1. Yes 2. No 3. Don’t know |
| 220 | Hypertension can cause premature death, if left untreated. | 1. Yes 2. No 3. Don’t know |
| **E. Knowledge towards Hypertension treatment methods** | | |
| 221 | Herbal medications used to control hypertension. | 1. Yes 2. No 3. Don’t know |
| 222 | Chemical drugs used to control hypertension. | 1. Yes 2. No 3. Don’t know |
| 223 | Taking healthy diet used to control hypertension. | 1. Yes 2. No 3. Don’t know |
| 224 | Minimizing stress used to control hypertension. | 1. Yes 2. No 3. Don’t know |
| 225 | Smoking and alcohol cessation used to control hypertension. | 1. Yes 2. No 3. Don’t know |
| 226 | Performing regular exercise used to control hypertension. | 1. Yes 2. No 3. Don’t know |
| **Part III. Behavioral characteristics** | | |
| **A. Cigarette smoking** | | |
| 301 | Do you smoke cigarettes? | 1. Yes 2. No |
| **Note:** If your answer is **No** for question number 301 go to question number 303 | | |
| 302 | How frequently do you smoke? | 1. Daily 2. Once /wk 3. 2 -3 days/wk 4. 4-5 days/wks 5. Other specify---------------- |
| 303 | During the time of smoking how many cigarettes do you smoke? | 1. 1 or less cigarette 2. 2-5 cigarettes 3. 6-10 cigarettes 4. 11 and more cigarettes |
| **B. Khat chewing** | | |
| 304 | Do you have a habit of Khat chewing? | 1. Yes 2. No |
| **Note:** If your answer is **No** for question number 304 go to question number 306 | | |
| 305 | How frequently do you chewing the khat? | 1. Daily 2. Most week days 3. Weekends only 4. On occasions |
| **C. Alcohol drinking** | | |
| 306 | Do you drink alcohol? | 1. Yes 2. No |
| **Note:** If your answer is **No** for question number 306 go to question number 309 | | |
| 307 | How often do you take alcoholic drinks? | 1. Daily 2. 5-6 days per week 3. 1-4 days per week 4. 1-3 days per week 5. Other specify--------------- |
| 308 | On average, how much do you usually drink alcohol? (one portion of alcohol is having at least 1 glass of wine, 1 bottle of beer, a 50g of ouzo) | 1. Less than one drink 2. One to three drinks 3. Four to six drinks 4. Seven or more drinks |
| **D. Dietary history** | | |
| 309 | Do you eat fruits? | 1. Yes 2. No |
| **Note:** If your answer is **No** for question number 309 go to question number 312 | | |
| 310 | In a typical week, on how many days do you eat fruit? | 1. Daily 2. 1- 4 days per week |
| 311 | How many servings of fruits do you eat on one of those days? (1 serving= one orange/ apple/banana/peach/mango/grapes etc). | 1. 1- 4 serving of fruits 2. 5 or more servings of fruits |
| 312 | Do you eat vegetables? | 1. Yes 2. No |
| **Note:** If your answer is **No** for question number 312 go to question number 315 | | |
| 313 | In a typical week, on how many days do you eat vegetables? | 1. Daily 2. 1- 4 days per week |
| 314 | How many servings of vegetables do you eat on one of those days? (1 serving= three tablespoons of cooked vegetables) | 1. 1- 4 serving of vegetables 2. 5 or more servings of vegetables |
| **E. Physical activity** | | |
| 315 | Do you perform regular physical exercise? | 1. Yes 2. No |
| **Note:** If your answer is **No** for question number 315 go to question number 401 | | |
| 316 | What type of exercise do you perform? | 1. Walking 2. Jogging 3. Cycling 4. Swimming |
| 317 | How often do you exercise? | 1. < 5 days per week 2. ≥ 5 days per week |
| 318 | For how many minutes do you exercise per session? | 1. <30 minutes 2. ≥30 minutes |
| **Part IV. History of chronic illness** | | |
| 401 | Do you have family history of hypertension? | 1. Yes 2. No 3. I don’t know |
| **Note:** If your answer is **No or Don’t know** for question number 401 go to question number 403 | | |
| 402 | Who is your family? | 1. Father 2. Mother 3. Grand father 4. Grand mother |
| 403 | Have you ever been told by a doctor that you have diabetes mellitus? | 1. Yes 2. No |
| 404 | Have you ever been told by a doctor that you have cardiovascular problem? | 1. Yes 2. No |
| 405 | Have you ever been told by a doctor that you have kidney problem? | 1. Yes 2. No |
| **Part V. Body Mass Index and Blood Pressure level** | | |
| **A. Height and Weight** | | |
| 501 | Weight | --------------------------kg |
| 502 | Height | --------------------------Meter |
| 503 | BMI level | -------------------------kg/m^2^ |
| **B. Blood Pressure level** | | |
| 504 | First blood pressure measurement | Systolic--------------mmHg |
|  |  | Diastolic------------ mmHg |
| 505 | Second blood pressure | Systolic--------------mmHg |
|  |  | Diastolic-------------mmHg |
| 506 | Average blood pressure | Systolic--------------mmHg |
|  |  | Diastolic-------------mmHg |

WE THANK YOU FOR THE FULL COOPRERATION!

SUPPERVISOR NAME------------------------- SIGNATURE--------------DATE----------------

**መጠይቆች**

በባህርዳር ከተማ ባንክ ቤቶች ላይ በሚሰሩ ሰራተኞች ላይ ያልተመረመረ የደም ግፊት ያለዉን የስርጭት መጠን እና ተዛማጅ ችግሮችን ለመለየት የቀረበ መጠይቅ 2012 ዓ.ም

ጥናትና ምርምሩን የሚያካሂደው ሰው ሙሉ ስም: ----------------------------------ፊርማ-------ቀን-----------

| **መመሪያ ፡**-ጥያቄዎቹን አንብበዉ ከተረዱ በኋላ መልስዎን ያክብቡ | | |
| --- | --- | --- |
| **ክፍል 1. የስነ ሕዝብ እና ማህበራዊ ጉዳዮች** | | |
| **ተራ**  **ቁጥር** | **ጥያቄ** | **አማራጭ መልስ** |
| 101 | እድሜ | -----------አመት |
| 102 | ፆታ | 1. ወንድ 2. ሴት |
| 103 | የጋብቻ ሁኔታ | 1. ያላገባ /ያላገባች 2. ያገባ/ያገባች 3. የፈታ/የፈታች 4. የሞተችበት/የሞተባት |
| 104 | ሐይማኖት | 1. ኦርቶዶክስ ክርስቲያን 2. ሙስሊም 3. ካቶሊክ 4. ፕሮቴስታንት 5. ሌላ ካለ ይጥቀሱ----------------- |
| 105 | የትምህርት ደረጃ | -------------------------- |
| 106 | በዚህ ባንክ ቤት ውስጥ ያለዎት የስራ ድርሻ ምንድን ነዉ? | ------------------------- |
| 107 | የአገልግሎት ዘመን | -----------------------ዓመት |
| **ክፍል 2. ስለደም ግፊት ያለዎት እዉቅና** | | |
| **ሀ. የደም ግፊትን ስለሚያመጡ ነገሮች ያለዎት እዉቅና** | | |
| 201 | ጨዉ የበዛበት ምግብ መመገብ ለደምግፊት ያጋልጣል፡፡ | 1. ያጋልጣል 2. አያጋልጥም 3. አላዉቅም |
| 202 | ከልክ በላይ የሆነ ዉፍረት ለደም ግፊት ያጋልጣል፡፡ | 1. ያጋልጣል 2. አያጋልጥም 3. አላዉቅም |
| 203 | ከልክ በላይ የሆነ ጭንቀት ወይም ብስጭት ለደም ግፊት ያጋልጣል፡፡ | 1. ያጋልጣል 2. አያጋልጥም 3. አላዉቅም |
| 204 | ከልክ በላይ የሆነ አልኮል መጠጣት ለደም ግፊት ያጋልጣል፡፡ | 1. ያጋልጣል 2. አያጋልጥም 3. አላዉቅም |
| 205 | ሲጋራ ማጨስ ለደም ግፊት ያጋልጣል፡፡ | 1. ያጋልጣል 2. አያጋልጥም 3. አላዉቅም |
| 206 | የደም ግፊት በዘር ይተላለፋል፡፡ | 1. ይተላለፋል 2. አይተላለፍም 3. አላዉቅም |
| **ለ. የደም ግፊት በሽታ ስለሚያሳያቸዉ ስሜቶች እና ምልክቶች ያለዎት እዉቅና** | | |
| 207 | የራስ ምታት አንዱ የደም ግፊት በሽታ ስሜት ነዉ፡፡ | 1. ነዉ 2. አይደለም 3. አላዉቅም |
| 208 | ራስ ማዞር አንዱ የደም ግፊት በሽታ ስሜት ነዉ፡፡ | 1. ነዉ 2. አይደለም 3. አላዉቅም |
| 209 | የትንፋሽ ማጠር አንዱ የደም ግፊት በሽታ ስሜት እና ምልክት ነዉ፡፡ | 1. ነዉ 2. አይደለም 3. አላዉቅም |
| 210 | ፈጣን የልብ ምት አንዱ የደም ግፊት በሽታ ስሜት እና ምልክት ነዉ፡፡ | 1. ነዉ 2. አይደለም 3. አላዉቅም |
| **ሐ. ስለ ደም ግፊት መከላከያ ዘዴዎች ያለዎት እዉቅና** | | |
| 211 | ተከታታይ የሆነ የአካል ብቃት እንቅስቃሴ ማድረግ የደም ግፊትን ይከላከላል፡፡ | 1. ይከላከላል 2. አይከላከልም 3. አላዉቅም |
| 212 | የደም ግፊት ምርመራ ማድረግ ከፍተኛ የሆነ የደምግፊትን ለመከላከል ይጠቅማል፡፡ | 1. ይጠቅማል 2. አይጠቅምም 3. አላዉቅም |
| 213 | ጭንቀትን መቀነስ የደም ግፊትን ይከላከላል፡፡ | 1. ይከላከላል 2. አይከላከልም 3. አላዉቅም |
| 214 | አትክልት እና ፍራፍሬ አዘዉትሮ መመገብ የደም ግፊትን ይካለከላል፡፡ | 1. ይከላከላል 2. አይከላከልም 3. አላዉቅም |
| 215 | ጨዉ የበዛበት ምግብ አለመመገብ የደም ግፊትን ይከላከላል፡፡ | 1. ይከላከላል 2. አይከላከልም 3. አላዉቅም |
| **መ. የደም ግፊት ስለሚያመጣዉ የጎንዮሽ ጉዳት ያለዎት እዉቅና** | | |
| 216 | የደም ግፊት ካልታከመ የልብ በሽታ ያመጣል፡፡ | 1. ያመጣል 2. አያመጣም 3. አላዉቅም |
| 217 | የደም ግፊት ካልታከመ የእይታ ችግር ያመጣል፡፡ | 1. ያመጣል 2. አያመጣም 3. አላዉቅም |
| 218 | የደም ግፊት ካልታከመ የአንጎል ጥቃት ያመጣል፡፡ | 1. ያመጣል 2. አያመጣም 3. አላዉቅም |
| 219 | የደም ግፊት ካልታከመ የኩላሊት ስራ ማቆም ያመጣል፡፡ | 1. ያመጣል 2. አያመጣም 3. አላዉቅም |
| 220 | የደም ግፊት ካልታከመ ሞት ያመጣል፡፡ | 1. ያመጣል 2. አያመጣም 3. አላዉቅም |
| **ሠ. ስለ ደም ግፊት የህክምና ዘዴዎች ያለዎት እዉቅና** | | |
| 221 | የባህል መድሀኒት መዉሰድ የደም ግፊትን ለመቆጣጠር ይጠቅማል:: | 1. ይጠቅማል 2. አይጠቅምም 3. አላዉቅም |
| 222 | የኬሚካል መድሀኒት መዉሰድ የደም ግፊትን ለመቆጣጠር ይጠቅማል:: | 1. ይጠቅማል 2. አይጠቅምም 3. አላዉቅም |
| 223 | ጤናማ የሆነ ምግብ መመገብ የደም ግፊትን ለመቆጣጠር ይጠቅማል፡፡ | 1. ይጠቅማል 2. አይጠቅምም 3. አላዉቅም |
| 224 | ጭንቀትን መቀነስ የደም ግፊትን ለመቆጣጠር ይጠቅማል፡፡ | 1. ይጠቅማል 2. አይጠቅምም 3. አላዉቅም |
| 225 | ሲጋራ እና አልኮል ማቆም የደም ግፊትን ለመቆጣጠር ይጠቅማል፡፡ | 1. ይጠቅማል 2. አይጠቅምም 3. አላዉቅም |
| 226 | ተከታታይ የሆነ የአካል ብቃት እንቅስቃሴ ማድረግ የደም ግፊትን ለመቆጣጠር ይጠቅማል፡፡ | 1. ይጠቅማል 2. አይጠቅምም 3. አላዉቅም |
| **ክፍል 3. የአኗኗር ዘይቤ** | | |
| **ሀ. ሲጋራ ማጨስ** | | |
| 301 | ሲጋራ ያጨሳሉ? | 1. አዎ 2. አላጨስም |
| **መመሪያ፡** ለጥያቄ ቁጥር 301 መልሰወ አላጨስም ከሆነ ወደ ጥያቄ ቁጥር 304 ይቀጥሉ | | |
| 302 | በየስንት ጊዜው ያጨሳሉ? | 1. በየቀኑ 2. በሳምንት አንድ ጊዜ 3. በሳምንት ከ 2-3 ቀን 4. በሳምንት ከ 4-5 ቀን 5. ሌላ ካለ ይጥቀሱ----------------- |
| 303 | በሚያጨሱበት ሰአት ምን ያህል ሲጋራ ያጨሳሉ? | 1. 1 እና ከዚያ በታች 2. ከ 2-5 ሲጋራ 3. ከ 6-10 ሲጋራ 4. 11 እና ከዚያ በላይ |
| **ለ. ጫት መቃም** | | |
| 304 | ጫት የመቃም ልምድ አለዎት? | 1. አዎ 2. የለኝም |
| **መመሪያ፡** ለጥያቄ ቁጥር 304 መልስወ የለኝም ከሆነ ወደ ጥያቄ ቁጥር 306 ይቀጥሉ | | |
| 305 | በየስንት ጊዜው ይቅማሉ? | 1. በየቀኑ 2. አብዛኛዉን የሳምንት ቀናት 3. በየሳምንቱ 4. አልፎ አልፎ |
| **ሐ. የአልኮል መጠጥ** | | |
| 306 | የአልኮል መጠጥ ይጠጣሉ? | 1. አዎ 2. አልጠጣም |
| **መመሪያ፡** ለጥያቄ ቁጥር 306 መልስወ አልጠጣም ከሆነ ወደ ጥያቄ ቁጥር 309 ይቀጥሉ | | |
| 307 | በየስንት ጊዜው ይጠጣሉ? | 1. በየቀኑ 2. በሳምንት ከ 5-6 ቀን 3. በሳምንት ከ 1-4 ቀን 4. በሳምንት ከ 1-3 ቀን 5. ሌላ ካለ ይጥቀሱ----------------- |
| 308 | በአማካይ ምን ያህል ይጠጣሉ? መገለጫ፡- አንድ መጠን የሚባለዉ ቢያንስ 1ብርጭቆ ወይን፣ 1ጠርሙስ ቢራ፣ ወይም 50 ሲሲ ኡዞ፣ ጂን የመሳሰሉ አልኮሎች ማለት ነዉ፡፡ | 1. አንድ መጠን 2. ከ1-3 መጠን 3. ከ 4-6 መጠን 4. ከ7 መጠን በላይ |
| **መ.** **የአመጋገብ ታሪክ** | | |
| 309 | ፍራፍሬ ይመገባሉ? | 1. አዎ 2. አልመገብም |
| **መመሪያ፡** ለጥያቄ ቁጥር 309 መልስወ አልመገብም ከሆነ ወደ ጥያቄ ቁጥር 312 ይቀጥሉ | | |
| 310 | በሳምንት ምን ያህል ቀን ይመገባሉ? | 1. በየቀኑ 2. በሳምንት ከ 1-4 ቀን |
| 311 | በሚመገቡበት ሰአት ስንት የፍራፍሬ አይነት ይመገባሉ?  ( አንድ የፍራፍሬ አይነት= አንድ ብርቱካን/አፕል/ሙዝ/ ዎይን/ኮክ እና የመሳሰሉት). | 1. ከ1- 4 የፍራፍሬ አይነት 2. 5 እና ከዚያ በላይ የፍራፍሬ አይነት |
| 312 | አትክልት ይመገባሉ? | 1. አዎ 2. አልመገብም |
| **መመሪያ፡** ለጥያቄ ቁጥር 312 መልስወ አልመገብም ከሆነ ወደ ጥያቄ ቁጥር 315 ይቀጥሉ | | |
| 313 | በሳምንት ምን ያህል ቀን ይመገባሉ? | 1. በየቀኑ 2. በሳምንት ከ 1-4 ቀን |
| 314 | በሚመገቡበት ሰአት ስንት የአትክልት አይነት ይመገባሉ?  (አንድ የአትክልት አይነት=ሶስት የሾርባ ማንኪያ የተዘጋጁ አትክልቶች). | 1. ከ1- 4 የአትክልት አይነት 2. 5 እና ከዚያ በላይ የአትክልት አይነት |
| **ሠ. የአካል ብቃት ዕንቅስቃሴ** | | |
| 315 | የአካል ብቃት እንቅስቃሴ ያደርጋሉ? | 1. አዎ 2. አላደርግም |
| **መመሪያ፡** ለጥያቄ ቁጥር 315 መልስወ አላደርግም ከሆነ ወደ ጥያቄ ቁጥር 401 ይቀጥሉ | | |
| 316 | ምን አይነት እንቅስቃሴ ያደርጋሉ? | 1. እርምጃ 2. ሶምሶማ 3. ብስክሌት መንዳት 4. ዉሀ ዋና |
| 317 | በየስንት ጊዜዉ እንቅስቃሴ ያደርጋሉ? | 1. በሳምንት ከአምስት ቀን በታች 2. በሳምንት አምስት ቀን እና ከጊዜ በላይ |
| 318 | በሚንቀሳቀሱበት ጊዜ ለምን ያህል ደቂቃ ይንቀሳቀሳሉ? | 1. ከ30 ደቂቃ በታች 2. 30 ደቂቃ እና ከዚያ በላይ |
| **ክፍል 4. ካሁን በፊት** **የታዎቁ በሽታዎች** | | |
| 401 | የደም ግፉት በሽታ በቤተሰብዎ አለበወት? | 1. አዎ 2. የለብኝም 3. አላዉቅም |
| **መመሪያ፡** ለጥያቄ ቁጥር 401 መልስወ የለብኝም ወይም አላዉቅም ከሆነ ወደ ጥያቄ ቁጥር 403 ይቀጥሉ | | |
| 402 | ቤተሰብዎት ማን ነዉ? | 1. አባት 2. እናት 3. ወንድ አያት 4. ሴት አያት |
| 403 | እርሰዎ በህክምና የተረጋገጠ የስኳር በሽታ አለብዎት? | 1. አዎ 2. የለብኝም |
| 404 | እርሰዎ በህክምና የተረጋገጠ የልብ በሽታ አለብዎት? | 1. አዎ 2. የለብኝም |
| 405 | እርሰዎ በህክምና የተረጋገጠ የኩላሊት በሽታ አለብዎት? | 1. አዎ 2. የለብኝም |
| **ክፍል 5. የሰዉነት አቋም መጠን እና የደም ግፊት መጠን ልኬታ** | | |
| **ሀ. የክብደት እና የቁመት መጠን** | | |
| 501 | ክብደት | -----------------------ኪሎ ግራም |
| 502 | ቁመት | ------------------------ሜትር |
| 503 | የሰዉነት አቋም መጠን | ------------------------ኪ.ግ/ሜ^2^ |
| **ለ. የደም ግፊት መጠን** | | |
| 504 | የመጀመሪያው የደም ግፊት መጠን | ሲስቶሊክ---------ሚሊ ሜትር ሜርኩር |
|  |  | ዲያስቶሊክ--------ሚሊ ሜትር ሜርኩር |
| 505 | ሁለተኛ የደም ግፊት መጠን | ሲስቶሊክ---------ሚሊ ሜትር ሜርኩር |
|  |  | ዲያስቶሊክ--------ሚሊ ሜትር ሜርኩር |
| 506 | አማካኝ የደም ግፊት መጠን | ሲስቶሊክ---------ሚሊ ሜትር ሜርኩር |
|  |  | ዲያስቶሊክ--------ሚሊ ሜትር ሜርኩር |

ላደረጉልኝ ሙሉ ተሳትፎ በጣም አመሰግናለሁ

መጠየቁ በትክክል መሞላቱን የተከታተለው ሰው ስም ----------------------------ፊ ር ማ -------ቀን ---------
