# Supplementary material for: Undiagnosed hypertension and associated factors among bank workers in Bahir Dar City, Northwest, Ethiopia, 2020. A cross-sectional study
Source: PLoS One. 2021 May 27;16(5):e0252298. doi: 10.1371/journal.pone.0252298 (PMC8158901; doi:10.1371/journal.pone.0252298)
Supplement: Attachment — Submitted filename: Response to Reviewers.docx [file pone.0252298.s003.docx]

**Responses to the reviewers’ comments**

Title of the article: **Undiagnosed Hypertension and Associated Factors Among Bank Workers in Bahir Dar City, Northwest, Ethiopia, 2020. A Cross-Sectional Study.**

**Dear, reviewers and editors**

In the first place, I thank you very much for your great, constructive and detail comments to improve the quality of our paper.

Based on your following comments, the corresponding corrections were done and/ or reflections were given:

|  | **Comments** | **Response** |
| --- | --- | --- |
| **SN** | **REVIEWER COMMENTS** |  |
| **1** | Please ensure that your manuscript meets PLOS ONE's style requirements, including those for file naming. | Thank you for your comments. Yes, you are right there is some fallacy with PLOS ONE's style requirements. I have made modifications according to the PLOS ONE’s style. I made titles and sub titles heading 1, 2 and 3 and the fonts are corrected as per PLOS ONE style throughout the document. |
| **2** | Please include additional information regarding the survey or questionnaire used in the study and ensure that you have provided sufficient details that others could replicate the analyses. For instance, if you developed a questionnaire as part of this study and it is not under a copyright more restrictive than CC-BY, please include a copy, in both the original language and English, as Supporting Information. If the original language is written in non-Latin characters, for example Amharic, Chinese, or Korean, please use a file format that ensures these characters are visible. | Thank you for the comment, we included additional information regarding the questionnaire. Page7, line127-130  We didn’t develop the questionnaire but we adapted from previous literatures and we put the references. Page 7, line 130  The questionnaire is not under a copy right and hence, we include the copy of it in both its original language (English) and that we translated in to Amharic. |
| **3** | Please state whether you validated the questionnaire prior to testing on study participants. Please provide details regarding the validation group within the methods section. | Thanks, Pretest was done before the actual data collection as we stated in Page 8,Line 149  But detail validation was not done because we adapted it from previous similar literatures including studies conducted in Ethiopia. And hence, we supposed as it valid for our study. |
| **4** | In your Methods section, please provide additional information about the participant recruitment method and the demographic details of your participants. Please ensure you have provided sufficient details to replicate the analyses such as:  -a description of any inclusion/exclusion criteria that were applied to participant recruitment,  -a statement as to whether your sample can be considered representative of a larger population,  -a description of how participants were recruited, and  -Descriptions of where participants were recruited and where the research took place. | Thank you for your comment. We have added information’s related to participant recruitment method, inclusion/exclusion criteria, where participants were recruited and where the research took place. Bank workers who were working in the selected banks and available during the time of data collection and those who were not diagnosed as hypertensive and/or use of anti-hypertensive medications were included from the study. In other words, those who were diagnosed as hypertensive and/or use of anti-hypertensive medications were excluded from the study. In addition to Pregnant bank workers and Janitors. Page 6, line 114-122  We believe the sample size can be representative for the larger population which means for our source population that our sample is drew. Because our sample size is more than 30% of the source population and 50% of banks were selected for the study. |
| **5** | We note that you have indicated that data from this study are available upon request. PLOS only allows data to be available upon request if there are legal or ethical restrictions on sharing data publicly. For information on unacceptable data access restrictions, please see http://journals.plos.org/plosone/s/data-availability#loc-unacceptable-data-access-restrictions.  In your revised cover letter, please address the following prompts:  a) If there are ethical or legal restrictions on sharing a de-identified data set, please explain them in detail (e.g., data contain potentially identifying or sensitive patient information) and who has imposed them (e.g., an ethics committee). Please also provide contact information for a data access committee, ethics committee, or other institutional body to which data requests may be sent.  b) If there are no restrictions, please upload the minimal anonymzed data set necessary to replicate your study findings as either Supporting Information files or to a stable, public repository and provide us with the relevant URLs, DOIs, or accession numbers. Please see http://www.bmj.com/content/340/bmj.c181.long for guidelines on how to de-identify and prepare clinical data for publication. For a list of acceptable repositories, please see http://journals.plos.org/plosone/s/data-availability#loc-recommended-repositories. | Thank you, there are no restrictions and we uploaded the full data as additional file. |
|  |  |  |
| **7** | Thank you for stating the following beneath the Acknowledgments Section of your manuscript:  'Funding  This study was supported by Amhara Regional Health Bureau.'  We note that you have provided funding information that is not currently declared in your Funding Statement. However, funding information should not appear in the Acknowledgments section or other areas of your manuscript. We will only publish funding information present in the Funding Statement section of the online submission form.  a. Please remove any funding-related text from the manuscript and let us know how you would like to update your Funding Statement. Currently, your Funding Statement reads as follows:  'No. The funders had no role in study design, data collection and analysis, decision to publish, or preparation of the manuscript'  b. Please include your amended statements within your cover letter; we will change the online submission form on your behalf. | Thank you for your comment. We have removed the funding-related text from the manuscript. And I Kindly request you to update my funding statement on the behalf of me. Please change it from ‘’no’’ to ‘’Yes’’. The amended statement is “This research was funded by Amhara regional health bureau’’  We included our Amended statement in our cover letter. |
| 8 | Abstract  • Specify that study was conducted in Ethiopia  • Specify the statistical approach that was used for the analysis in the last sentence of the methods section  • What percentage of participants had diagnosed hypertension?  • Round up the adjusted odds ratios and confidence intervals to 2 decimal places. | Thank you for your comment.  We specified as a study was conducted in Ethiopia. Page 2, Line 31  We Specified the statistical approach that was used for the analysis. Page 2, Line 36-37  Participants who were diagnosed as hypertensive using anti-hypertensive medications were excluded in this study so, we have not the data about percentage of diagnosed hypertension. It may be other research topic/objective.  We have rounded up the adjusted odds ratio and confidence interval to 2 decimal places as per your comment. Page 2, line 43-47 |
| 9 | Introduction  • Page 4, line 75, revise sentence from “In the year of 2007” to “In 2007”  • Line 90 and 89 are redundant. Both sentences can be combined to make the point more concise | Thank you, we have edited as per your comments. Page 4, Line 76  We have combined the statements. Page 5, line 89-90 |
| 10 | The study participants  • Change the subheading to Participants  • Provide range or approximate number of bank workers to provide sufficient information on the source population. | Thank you for your comment.  We have changed the headings and We provided number of bank workers that can provide sufficient information. Page 6, line 113-114 |
| 11 | Sample size and procedure  • The formula for the sample size calculation is not needed in this section. Table 1 is also not necessary.  • Reference should be provided for WHO classification | Thank you for your comment. We have removed the sample size calculation formula.  We provided reference fro WHO classification of body mass index. Page 7 , Line 147 |
| 12 | Measurement  • The first sentence should be revised and the correct description should be used to describe the American Heart Association Hypertension Clinical Guidelines. A citation should be provided after the first sentence.  • The last sentence is unclear. What guideline is being referred to in this context? | We revised the first sentence  We tried to describe the American Heart Association Hypertension Clinical Guidelines correctly. Page 8, Line 154-156  The citation is provided for the first sentences  We modified the last sentences page 8, Line 166 |
| 13 | Data quality control  • This section can be integrated into the other sections and shortened.  • There is a typo in line 183 “questioner” | Yes, we accepted your comment and we have integrated it with subheading of Data collection. Page 8, Line 149-153  We have removed the word questioner when we merge the sub heading Data quality control. |
| 14 | Statistical Analysis  • Add description | Thank you for your comment. We have added some descriptions in the statistical analysis section. Page 8 , Line 169-170 |
| 15 | Results  • Line 222: add a description of chat for readers who are not familiar with that terminology  • There are several grammatical errors that need to be corrected.  • In Table 3, the number of drinks should be presented separately for men and women because of the differences in recommendation.  • Add footnote should be added to Table 3 to provide a description of all the abbreviations used in Table 3.  • All Odds Ratios and 95% CIs should be reported to two decimal places.  • The proportion of participants who had normal blood pressure, elevated blood pressure, stage 1 and stage 2 hypertension are not reported. | Thank you for your comments.  I have changed chat to khat which is more familiar terminology. Page 10, Line 198  We tried to correct grammatical errors. Page 2,4,5,6,8,9,13,15, Line, 41,42, 81,91,118, 162, 166,167, 180, 233,235, 251  We presented separately the number of drinks for men and women. Table 2. Page 11  We added footnote to table 3. Page 11. Now it is changed to table 2  We rounded up All Odds Ratios and 95% CIs to two decimal places Table 4. Page 14. Now it is changed to table 3  We reported the proportion as per your comment. Page 12, line 216-218 |
| 16 | On the introduction, the reason for choosing bank workers from the general population for the study should be emphasized. | Thank you, we added the reasons why we choose bank workers for this study in the introduction section. Page 5,line 98-102 |
| 17 | On the study design and period section, the authors have mentioned that there are 2 government and 16 private banks in Bahir Dar, how many branches were included in the study? What was your selection criteria? | Thank you for your comment.  9 Banks out of 18 were included in this study.  They were selected by lottery method  We clearly explained about it under the subheading of Participants. Page 6, Line 113 |
| 18 | The authors use P value from a study conducted in the general population in Gulelle sub-city of Addis Ababa while your study was focused on a specific segment of the population. How do you explain this? | Thank you for your comment.  In Ethiopia there is no study conducted among bank workers on this topic. We didn’t have option other than using the P value of the study conducted on the general population. We assumed taking P value of the study conducted on the general population is better than taking P value as 50%. Actually it can be the limitation of this study. |
| 19 | Line 169-174  “Level of blood pressure is classified as Normal; systolic blood pressure <120 and diastolic blood pressure <80 mm Hg, Elevated; systolic blood pressure 120-129 mm Hg and diastolic blood pressure <80 mm Hg, Hypertension: stage 1; systolic blood pressure 130-139 mm Hg or diastolic blood pressure 80-89 mm Hg and Hypertension: stage 2; systolic blood pressure =140 mm Hg or diastolic blood pressure =90 mm Hg”. Such classification is not used in the result section. Better to present information in a uniform manner. | We included the report in result section. Please see the response given in number 15. |
| 20 | The STEPS manual have an updated version as of 26 January 2017, why did you stick to the older version? | When we revise different literatures, we got that a lot of literatures are used the older version and we used as it is. This is our mistake and it is also can be another limitation of the study. |
| 21 | The P-value cutoff to declare significance mentioned in the methodology section is inconsistent with that in the result section. | Thank you for your comment. This is typing error so; we rewrite the P-value cutoff point. Page 9, line 173. |
| 22 | Table 2: Marital status…….check for percentage. | Thank you for your comment.  We have edited the percentage of marital status. Table1. Page 10, |
| 23 | Table 3: the percentage for drinking amount, frequency of eating fruit, serving of fruits, frequency of eating vegetables, serving of vegetables, exercise frequency, exercise duration should be calculated out of 100%. | I thank you for your comment.  We calculated them out of 100%. Page 11, Table 2 |
| 24 | Factors associated with undiagnosed hypertension: in the logistic regression analysis, how did you code for “yes” and “no” of undiagnosed hypertension? As there is a miss match between the COR and the frequency placed under each variable. | I thank you for your comment.  It was coded zero for no and one for yes.  We checked for miss mismatched. |
| 25 | Can we categorize the age group of 35-44 in the old age group? | I thank you for your comment. You are right, we have edited the statement. Page 16, line 277-178 |
