# Supplementary material for: Undiagnosed hypertension and associated factors among bank workers in Bahir Dar City, Northwest, Ethiopia, 2020. A cross-sectional study
Source: PLoS One. 2021 May 27;16(5):e0252298. doi: 10.1371/journal.pone.0252298 (PMC8158901; doi:10.1371/journal.pone.0252298)
Supplement: Attachment — Submitted filename: Response to Reviewers 28178.docx [file pone.0252298.s004.docx]

**Responses to the reviewers’ comments**

Title of the article: **Undiagnosed Hypertension and Associated Factors Among Bank Workers in Bahir Dar City, Northwest, Ethiopia, 2020. A Cross-Sectional Study.**

**Dear, reviewers and editors**

In the first place, I thank you very much for your great, constructive and detail comments to improve the quality of our paper.

Based on your following comments, the corresponding corrections were done and/ or reflections were given:

|  | **Comments** | **Response** |
| --- | --- | --- |
| **SN** | **REVIEWER COMMENTS** |  |
| **1** | Please review your reference list to ensure that it is complete and correct. If you have cited papers that have been retracted, please include the rationale for doing so in the manuscript text, or remove these references and replace them with relevant current references. Any changes to the reference list should be mentioned in the rebuttal letter that accompanies your revised manuscript. If you need to cite a retracted article, indicate the article’s retracted status in the References list and also include a citation and full reference for the retraction notice. | Thank you for your comment, I have reviewed the reference and it is complete and correct. Papers that have been retracted is not cited. |
| **2** | **Abstract**  Methods: line 37 “multiple logistic regression analysis was implemented” multiple should be replaced with multivariate. | Thank you for the comment, we replaced the word multiple with multivariate Page 2, line 37 |
| **3** | **Methods and materials**  Participants:  Line 121 better to replace study subjects with study participants. | Thanks, Study subjects is replaced by study participants. Page7,line142 |
| **4** | Line 124 it’s not clear whether the proportional allocation preceded or succeeded the selection of the bank workers by the random generator software.  Sample size calculation should not be deleted instead presented in a precise manner (I don’t think the recommendation of the previous reviewer was for you to delete the whole thing) | Thank you for your comment. We made a correction page 7, line 144  -Sample size calculation is presented in a precise manner. Page 6, line113-134 |
